# A Systematic Review of Perinatal Social Support Interventions for Asylum-seeking and Refugee Women Residing in Europe

**DOI:** 10.1007/s10903-021-01242-3

**Published:** 2021-07-17

**Authors:** Marie-Clare Balaam, Carol Kingdon, Melanie Haith-Cooper

**Affiliations:** 1 Research in Childbearing and Health (REACH) Unit, University of Central Lancashire, Preston, PR1 2HE Lancashire UK; 2 Faculty of Health Studies, University of Bradford, Bradford, BD7 1DP West Yorkshire UK

**Keywords:** Asylum-seeker, Refugee, Perinatal, Maternity, Social support

## Abstract

Asylum-seeking and refugee women currently residing in Europe face unique challenges in the perinatal period. A range of social support interventions have been developed to address these challenges. However, little is known about which women value and why. A critical interpretive synthesis was undertaken using peer reviewed and grey literature to explore the nature, context and impact of these perinatal social support interventions on the wellbeing of asylum-seeking and refugee women. Four types of interventions were identified which had varying impacts on women’s experiences. The impacts of the interventions were synthesised into five themes: Alleviation of being alone, Safety and trust, Practical knowledge and learning, being cared for and emotional support, and increased confidence in and beyond the intervention. The interventions which were most valued by women were those using a community-based befriending/peer support approach as these provided the most holistic approach to addressing women’s needs.

## Introduction

As part of wider global trends in international migration Europe has experienced an increased number of migrants moving into the region [1]. As part of this wider trend, and particularly since 2015, there has been an increase in the number of displaced people, including refugees and asylum-seekers within the numbers of those migrating to Europe. By the end of 2018 there were 2.4 million refugees and people in refugee-like situations and 860 thousand asylum-seekers in EU-27 States [2]. This increase in population has implications for the organisation of health and social care provision within the region.

Many asylum-seekers and refugees experience social and economic marginalisation as well as poor physical and mental health, with women, who make up half of the population of forced migrants [3], being identified as being particularly vulnerable. This situation is often exacerbated by pregnancy and early motherhood [2, 3]. Asylum-seeking and refugee women within Europe face challenges in accessing optimal maternity care, due to a range of issues including cost, communication, lack of health literacy and limited culturally appropriate care [4–7]. These challenges, exacerbated by the loss of the familial and social support structures women have in their home countries, mean that many women experience a lack social support and as a result face a range of significant psychosocial challenges to their maternal wellbeing [3, 6]. A combination of the increased number of refugee and asylum-seeking women of childbearing age within Europe [4, 8] and high fertility rates amongst migrant and refugee women [4, 9] has meant that European healthcare systems are struggling to provide appropriate and accessible maternity care for these women [4, 10, 11]. These challenges of provision, access to care and the impact of limited social support mean that asylum-seeking and refugee women experience poorer physical and mental wellbeing and poorer maternal and neonatal outcomes than other migrant women and non-migrant women [4, 6, 12].

One approach to addressing the poorer maternal wellbeing and psychosocial challenges faced by asylum-seeking and refugee women in the perinatal period, based on work done with other marginalised women, has been through providing interventions to increase social support. Research suggests that the maternal wellbeing of asylum-seeking and refugee women can be improved by interventions which seek to enhance and develop their levels of social support [13–16]. However, there is a lack of in-depth work considering what specific types of interventions women themselves identify as valuable and which type of interventions would be most effective in promoting wellbeing in different European national, social, political and cultural settings [13, 17].

This article addresses this gap in knowledge by presenting a Critical Interpretive Synthesis of literature which examines the provision, nature and impact of social support interventions offered to promote the wellbeing of asylum-seeking and refugee women in the perinatal period across Europe. Three questions were used to guide the review. First what social support interventions have been or are currently offered to asylum seeking and refugee women in Europe in the perinatal period? Secondly, how are these interventions located within the wider socio-cultural-political position of asylum-seekers and refugees in these countries and their maternity care settings and finally, what is the impact of these interventions on the wellbeing of asylum-seeking and refugee women?

Throughout this document the terms refugees and asylum-seekers are defined using United Nations (UN) definitions as follows:

‘*Refugees are individuals who are outside their country of nationality owing to a well-founded fear of being persecuted for reasons of race, religion, nationality, membership of a particular social group or political opinion…and are seeking in accordance with international conventions refuge in another country’* [18].

‘*Asylum-seekers are individuals whose claims of refugee status have not been definitively decided by the country they seek refuge in*’ [19].

## Method

A Critical Interpretive Synthesis (CIS) was undertaken following the approach described by Salmon et al. [20] developed from Dixon-Woods [21]. Eight electronic bibliographic health and social science databases (EMBASE, Public Health Database, Social Science Citation Index, Social work Abstracts, Maternal and Infant Health, Academic Search Complete, CINHAL, Medline) were initially searched in 2018 and then the search was updated in 2020. The search terms used reflected previous research [22] which identified that the terminology around migrant women within healthcare literature is problematic and imprecise. To address this, in addition to the key terms asylum-seeking and refugee, the following terms were included migrant, immigrant, undocumented, irregular migrants, asylum seekers, transient, refugee, foreign, illegal, alien and any literature recovered was then reviewed to ascertain if asylum seeking and refugee women were included within these broader terms. In addition to the electronic database search described above a grey literature search strategy comprised of four elements was undertaken including searches of; specific grey literature databases, websites of relevant organisations, Google and contacting relevant experts.

The results of the search were initially screened by abstract and then full text and duplicates removed. The inclusion criteria were papers that; presented primary research (qualitative, quantitative or mixed method); focused on social support interventions for/including asylum-seeking and/or refugee women based in Europe; were written in English; peer reviewed papers or grey literature and from any time period. All studies were appraised for quality using a schema devised by Wallace [23]. In line with a CIS approach this was used to assess the nature of the data rather than as a criterion for inclusion or exclusion.

Analysis of qualitative studies was undertaken using the approach outlined by Salmon [20]; a detailed reading of all included studies to allow for familiarisation and identification of themes, a process of constant comparison and exploration of constructs developed from original ideas in the papers as well as new synthetic constructs. The data were coded to identify patterns. Along with an ongoing critique of the nature of the literature as well as its content, new themes and original constructions in the papers were integrated into a theoretical framework bringing in wider literature and theories relevant to the topic. Quantitative papers and the quantitative aspects of mixed method papers were summarised narratively. Heterogeneity of quantitative outcomes prohibited meta-analyses of outcomes.

## Results

**Fig. 1:**
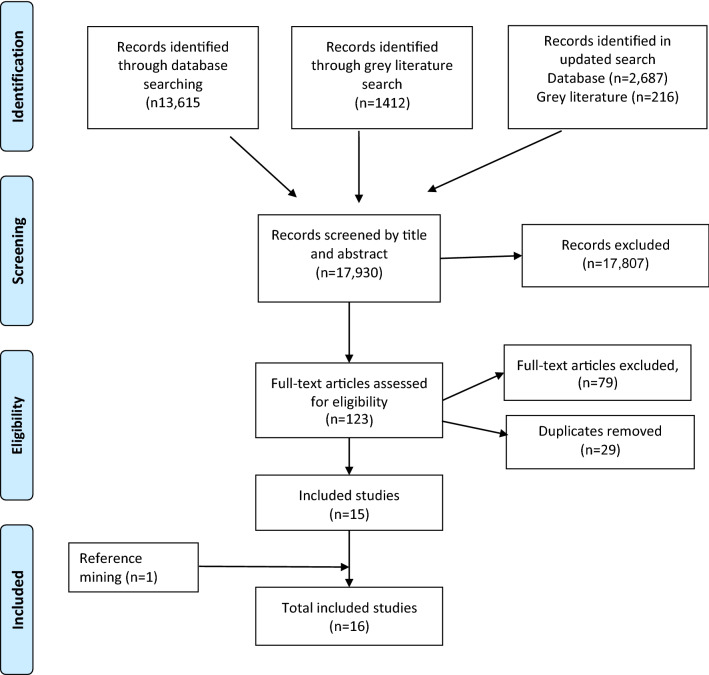
PRISMA diagram : updated and combined search 2020

Sixteen studies met the inclusion criteria. Nine were identified from the database search, six from the grey literature search and one from reference mining. See Fig. 1: PRISMA Diagram.

The selected studies were published between 1992 and 2020 in the UK (n = 8), Italy/Austria (n = 1), Netherlands (n = 3), Norway (n = 1), Sweden (n = 2) and one multi-site intervention (UK, Netherlands, Greece) (n = 1) and included both peer reviewed and grey literature. The studies included qualitative, quantitative and mixed methods approaches and ranged in size from 10 to 4000 participants. See Table 1.

**Table 1:** Summary of included study characteristics

Author and date	Country	Period	Characteristics of participants	Number in intervention	Number in study	Method	Quality Assessment (1–10)
Rocheron Y, Dickinson R, 1990	UK	Antenatal	Asian women	16 District health authorities (DHA)	Women in 3 DHAs who had/had not had link workers total = 100	Mixed methods	8
Parsons L, Day S, 1992	UK	Antenatal	Non-English-speaking women (identified by Asian & Turkish surnames)	Total 4000, 1000 in intervention, 3000 in 3 control groups	Total 4000, 1000 in intervention, 3000 in 3 control groups	Quantitative; Retrospective study	9
Ahmed S et al., 2006	UK	Postnatal	Bangladeshi women	194 supported in total, 80 by support worker alone	15 women	Mixed methods; questionnaires and interviews	9
Karl-Trummer U et al., 2006	Italy & Austria	Antenatal	Migrant/ethnic minority; Turkish, Indian & Pakistani women	Women 29 Austria, 12 Italy	Women 29 Austria, 12 Italy Staff 27 Italy, 5 Austria	Qualitative Interviews	3
Lederer J, 2009	UK	Antenatal, Postnatal	Socially excluded women—including asylum-seeking & refugee	46 women, 19 befrienders	15 women, 11 befrienders	Mixed methods; questionnaires, surveys & interviews	5
Akhavan S, Lundgren L, 2011	Sweden	Antenatal, Intrapartum, Postnatal	Immigrant women	25 midwives	10 midwives	Qualitative; semi structured interviews	9
Hesselink A E, Harting J, 2011	Netherlands	Antenatal	1st & 2nd generation Turkish women	119 women	119 women	Qualitative; process evaluation	9
Akhavan S, Edge D, 2012	Sweden	Antenatal, Intrapartum, Postnatal	Foreign born Non-European women	32 women	10 women	Qualitative; semi structured interviews	9
Hesselink A E et al., 2012	Netherlands	Antenatal	1st & 2nd generation Turkish women	119 in intervention, 120 in control	119 in intervention, 120 in control	Quantitative; non-randomised trail	8
O'Shaughnessy R, et al., 2012	UK	Postnatal	West African refugee & asylum-seeking women	unclear	57 women	Mixed methods; Participatory Action research	10
McCarthy R, Haith-Cooper M, 2013	UK	Antenatal, Postnatal	Asylum-seeking & refugee women	51 befrienders, 83 clients	51 befrienders, 83 clients	Qualitative; questionnaires, focus groups and interviews	8
Bhavnani V, Newburn M, 2014	UK	Antenatal, Postnatal	4 groups of women considered vulnerable/excluded communities, one of these groups was asylum-seeking & refugee women	Total volunteers = 299 volunteers in asylum-seeking & refugee groups = 65 Total women supported = 364 Asylum seeking & refugee women supported = 73	Total of 120 stakeholders in study Asylum-seeking & refugee women in study: 10 pre intervention questionnaire, 4 post intervention questionnaire, 5 interviews	Mixed methods; questionnaires, focus groups & interviews	7
Brookes H, Coster D, 2015	UK	Antenatal, Postnatal	Disadvantaged minority ethnic women including refugee and asylum-seeking women	Not given	14 parents (11 mums, 3 dads)	Qualitative: Semi-structured face-to-face interviews	7
Peters I A et al. 2017	Netherlands	Antenatal	Turkish & Moroccan women	262 women in control & 117 in intervention group	262 women in control & 117 in intervention group	Quantitative	9
Sioti E et al. 2019	Greece, UK, Netherlands	Antenatal, Postnatal	Migrant, Asylum-seeking and Refugee women (MAR)	Greece: Healthcare professionals (HCP) = 8, Maternity Peer support (MPS) = 6, women = 13 UK: HCP = 12, MPS = 14, MAR = 7, Netherlands: HCP = 5, MPS = 10, MAR = 12	Greece: Healthcare professionals (HCP) = 8, Maternity Peer support (MPS) = 4, MAR = 13 UK: HCP = 12, MPS = 14, MAR = 7, Netherlands: HCP = 5, MPS = 10, MAR = 12	Mixed methods; demographic & outcome data, Qualitative semi structured interviews & focus groups	7
Haugaard A. et al., 2020	Norway	Antenatal, Intrapartum, Postnatal	Newly arrived non-western migrant women	11 multicultural doulas	9 multicultural doulas	Qualitative; semi structured interviews	9

### Type, Timing and Context of Support in the Perinatal Period

Table 2 reports the type of support women received and the context of this support. Four categories of interventions were evident; (1) educational, (2) community befriending/peer support and community befriending plus, (3) community doula^1^ and (4) paraprofessional support.

**Table 2:** Summary of interventions and effectiveness

Author and date	Intervention	Intervention delivered by	Aim of intervention	Reported findings	Underlying rationale	Category of intervention	Impact/ themes
Rocheron Y, Dickinson R. 1990	Health promotion for Asian women, publicity campaign and link workers	Paid link workers	To encourage early ‘diagnosis’ of pregnancy, awareness and uptake of maternity care services and aid communication between professionals and Asian communities	Publicity campaign had limited success as failed to fully address the heterogeneity of the population. Link workers scheme improved women’s experiences	Deficiency/lack of cultural competence/communication	Paraprofessional	Gharib, Safety & Trust; being cared for
Parsons L, Day S. 1992	Health advocacy	Health advocates	To improve & facilitate communication between staff & local ethnic minority women, and to influence hospital policy & practise. The project grew from concerns over the quality of maternity care local women who did not speak English were receiving & the poor obstetric outcomes for women Asian backgrounds more generally	Improved outcomes amongst women who received support compared to control groups, including; reduction in length of antenatal stay, lower level of induction of labour & lower CS rates	Deficiency/lack of cultural competence/communication	Paraprofessional	Quantitative
Ahmed S et al. 2006	Bilingual peer support for breastfeeding	Bangladeshi support worker	To increase the uptake and duration of breastfeeding amongst Bangladeshi women through the employment of a Bangladeshi (bilingual) support worker provide extra breastfeeding support and education	The current system did not effectively support Bangladeshi women to breastfeed. The majority of women found support workers the most useful source of support for breastfeeding	Deficiency/lack of cultural competence/communication	Paraprofessional	Gharib, Safety & Trust; being cared for
Karl-Trummer U. et al. 2006	Prenatal courses for ethnic minority women	Health professionals	Health promotion intervention which emerged from the European Migrant friendly hospital initiative and the WHO ‘Making pregnancy safe initiative’ (2000) which identified poor outcomes amongst some migrant women	High efforts and good results, but low attendance	Clinical need/risk	Education	Gharib, Safety & Trust
Lederer J. 2009	Perinatal support project	Community volunteer befrienders	To improve the mental health of specific groups of women (one of these being AS&R women) considered to be vulnerable during pregnancy, to support increase maternal and child wellbeing	The intervention is reaching its target group, Users demonstrate reduced depression & anxiety, better social support & feel more confident as parents. Befrienders gained in confidence	Structural challenges	Community Befriending/peer support	Practical Learning and knowing about; I can do anything; Gharib, Safety & Trust; being cared for
Hesselink AE, Harting J. 2011	Antenatal education programme on smoking, infant care & psychosocial health for ethnic Turkish women	Turkish community health workers (CHW)	To provide antenatal education programme to prevent ‘unhealthy lifestyles, poor maternal infant care practises and poor psychosocial health in EM women. Specifically, Turkish women as identified as having an accumulation of identified risk factors e.g. smoking behaviour, depression, smothering, shaking and slapping	The intervention was effective in reaching a hard to reach population. The need crucial role of the community health workers and the need to integrate them into practises was noted. The intervention was, popular with mothers but had a mixed reception from staff	Clinical need/risk	Education	Gharib, Safety & Trust
Akhavan S, Lundgren I. 2011	Doula support for immigrant women	Voluntary community-based doula	To improve the quality of maternal health care for foreign born women, based upon the idea that the continuous support provided by a doula may reduce negative outcomes and allow foreign born women to receive the same level of care as non-foreign born women	Midwives described their experience of working with the doulas positively, but issues were raised about whether doulas should be used to compensate for deficiencies in the system or whether policies should be developed to address these deficiencies	Inequality /structural challenges	Community Doula	Gharib, Safety & Trust; being cared for
Akhavan S, Edge D. 2012	Community-Based Doulas for Foreign-Born Women's	Voluntary community-based doula	To improve the quality of maternal health care for foreign born women, based upon the idea that the continuous support provided by a doula may reduce negative outcomes and allow foreign born women to receive the same level of care as non-foreign-born women	Doulas can provide support & reassurance in the perinatal period & mean women have better care	Inequality/structural challenges	Community doula	Gharib, Safety & Trust, being cared for
Hesselink A E. et al. 2012	Happy Mother, Happy Baby (HMHB) antenatal education programme on smoking, infant care & psychosocial health for ethnic Turkish women	Turkish community health workers (CHW)	To provide antenatal education programme to prevent ‘unhealthy lifestyles, poor maternal infant care practises and poor psychosocial health in EM women. Specifically, Turkish women as identified as having an accumulation of identified risk factors e.g. smoking behaviour, depression, smothering, shaking and slapping	Improved knowledge about smoking, intention to engage in SIDS prevention & short-term SIDS prevention behaviour, no effect on smoking in pregnancy, long term SIDS prevention, soothing behaviour, serious depressive symptoms &parent- child attachment	Clinical need/risk	Education	Quantitative
O'Shaughnessy R. et al. 2012	Mental health service for asylum-seeking mothers and babies	Home Start volunteers & psychologists	To provide an early intervention mental health service focussing on supporting positive mother- baby relationships by delivering social & practical support as well as specialist therapeutic support	Women positively evaluated the intervention and there was an improvement in attachment relationships between mothers & their babies	Structural challenges	Community befriending/peer support plus	Practical Learning and knowing about; I can do anything; Gharib, Safety & Trust, being cared for
McCarthy R, Haith-Cooper M. 2013	Befriending for pregnant asylum-seeking and refugee women	Community Volunteer befrienders usually AS&R	To train volunteers to support pregnant AS&R women so they can understand and access timely maternity care & help them to connect with the local community & experience less social isolation	The project was beneficial to the women involved & had potential benefits to midwives and wider healthcare system. Befriending may help to meet the currently unmet needs of AS&R women	Structural challenges	Community befriending/peer support	Practical Learning and knowing about; I can do anything; Gharib, Safety & Trust; being cared for
Bhavnani V, Newburn M. 2014	Community perinatal support—community based outreach & 1 to 1 support for women in their homes or community venues	Community volunteer befrienders	To provide perinatal support & information to women and their partners who had been identified as vulnerable or from socially excluded communities	The intervention successfully reached the groups they had been aiming to reach. Demonstrated benefits for the parents and volunteers, provided lots of learning about working with diverse communities and about how their needs differ. Notes that AS&R women valued much more practical and informational/educational input as opposed to emotional support	Structural challenges	Community befriending/peer support	Practical Learning and knowing about; I can do anything; Gharib, Safety & Trust; being cared for
Brookes H, Coster D. 2015	Parental education programme for parents from a minority ethnic background	NSPCC staff	To support vulnerable parents in the transition to parenthood, focusing on the relationship between partners, the development of a positive parent-infant relationship and creating a social support network to help to develop inner resilience	The intervention provided relevant and useful information to the group targeted about maternity care, parenting practises and relationships	Structural challenges	Educational	Gharib, Safety & Trust
Peters I A et al. 2017	Use of culturally competent educational films about prenatal screening to increase knowledge amongst Fb women	Prenatal screening counsellors	To address research findings which identified low levels of IDM and low knowledge about PS amongst women from a non- western background	Intervention increased knowledge of and IDM around FS for foreign born women	Clinical need/risk	Education	Quantitative
Haugaard A et al. 2020	Multicultural doula support for newly arrived non western migrant women	Multicultural volunteer doula	The main purpose of the project was to strengthen, and ensure equitable access to, maternity care for migrant women through multicultural doulas	Multicultural doulas were an important resource during pregnancy and birth, for women & midwives. Their presence can strengthen maternity care for migrant women by providing information, ensuring continuity & building a cultural bridge between the migrant women and maternity care in Norway	Inequality/structural challenges	Community doula	Gharib, Safety & Trust, being cared for, Practical Learning and knowing about
Sioti E et al. 2019	An integrated, woman centred, culturally sensitive, and evidence-based approach to perinatal health care for migrant, asylum seeking or refugee (MAR) women	Multidisciplinary team of HCPs and volunteer community-based maternity peer supporters	To ensure a safe journey to motherhood, improve access and delivery of maternal healthcare for refugee and migrant women, and to improve maternal health equality within the European Union	The project was effective in providing access for MAR women to the usually hard-to-reach maternity health care services. This success can be attributed specifically to the involvement of the MPSs, who shared language and cultural background with the MAR mothers	Structural challenges	Community befriending/peer support	Gharib, Practical knowledge and learning, I can do anything, being cared for

Table 1 reports on the timing of these interventions. Six interventions take place only in the. antenatal period and 2 only in the postnatal period. 5 interventions offer support both antenatally and postnatally but not during the intrapartum period and 3 interventions offer support throughout the whole perinatal period including intrapartum. The most holistic support, support in the antenatal and postnatal periods and support offered throughout the who perinatal period (including intrapartum) is offered by the community based interventions offered by those interventions categorised as community befriending/peer support and community befriending plus and community doula.

### Impact of Interventions

Three studies provided exclusively quantitative data and six mixed methods studies reported some basic quantitative results. The quantitative data from these studies were of limited value for this research because it was not always possible to differentiate clearly between women who were/were not asylum-seekers and refugees who were involved in the intervention and the inclusion criteria in some studies were very imprecise. However, two quantitative studies [24, 25] reported some increased knowledge and/or behaviour change amongst participants related to prenatal screening, health promoting behaviours and parenting behaviours following the intervention. Also, one study reported the positive impact of paraprofessional health advocates on three maternal outcomes: reduced rates of caesarean section, and induction and shorter antenatal stay [26].

Due to the limited utility of the quantitative data from the included studies this paper focuses primarily on the qualitative data from the studies as this provides a way of considering the impact of the interventions on women’s experiences in the perinatal period and placing their experiences and voices at the centre of the study where possible.

From the qualitative data five themes emerged (detailed in Table 2 above) these were; (1) ‘Gharib’: alleviation of being alone, (2) ‘It’s only in this group you can ask’: safety and trust, (3) Practical knowledge and learning, (4) Being cared for and emotional support and (5) ‘I can do anything now’: increased confidence in and beyond the intervention.

### Gharib: Alleviation of being Alone

The first theme is encapsulated in an Arabic/Persian term Gharib used by Akhavan and Lundgren which they translated as;

*‘a feeling that one is alone, a stranger, and an outsider who cannot understand the languages, rules, laws and traditions in this new society'* [27].

Community befriending/peer support, community befriending plus and community doula type interventions all identified this sense of loneliness and isolation described by one woman as making her feel *‘really sad and hopeless’* [28]. These feelings were commonly linked to issues of poor communication and language difficulties and to a lack of understanding of how the new society functioned [27–32].

A number of studies described how women faced challenges communicating as they were unable to speak the language of the reception country [14, 27, 28, 32–34]. This led to, in the most extreme case, a woman who was unable to speak the language of the country and who had no translator, giving birth alone [14]. Interventions which provided befrienders or doulas who shared a common language allowed women to communicate more effectively reducing their sense of loneliness and isolation. One woman described how her befriender was the only person she had spoken to in her native tongue (Arabic) since arriving in the UK and how she ‘*took me out of my loneliness corner’* [14].

Several community befriending interventions reduced the sense of isolation felt by women by supporting them to access groups and group activities. Brookes and Coster [28], Bhavnani and Newburn [29] and O’ Shaughnessy et al. [30] all noted how accessing groups, spending time with other women and sharing experiences made women feel less alone and reduced their sense of isolation and feelings of stress. One woman noted that; *‘I think to share is very good …so this group – it means a lot*.’ [30] Another commented that;

*‘It’s a good thing to talk about. Because like when you are at home . . . I am alone with my baby . . . and when you see that letter [Home Office] . . . you are afraid. So if I come out of the house and meet people to talk about that. When I leave I will be a little bit better, So for stress, it’s good . . . it’s good to talk about that’* [30].

Staff noted that women faced challenges in understanding and accessing maternity care as they could not,

*‘speak the language, have been traumatised, isolated and are unaccustomed to the* system’ [34].

One woman explained; *‘I was lost, what should I do, when should I go to the hospital’* [34].

Several interventions, which provided doula or befriending support, were described by participants as acting as ‘*a bridge*’, supporting them as they dealt with new ideas and unfamiliar situations [28, 34]. Akhavan and Lundgren explained that the doulas in their interventions had a shared cultural background and this allowed them to.

*‘understand the women’s perception of life based experiences and, her customs and practises’* [27],

and support them as they sought to navigate settings they were unfamiliar with. In Haugaard’ s study the doulas believed that their shared migrant background allowed them to have extra empathy with their clients, one noted that.

*‘The midwives don’t have the same understanding as us. When we talk with the women, it is heart communication.’* [32]

### ‘It’s only in this Group you can ask’; Safety and Trust

A second theme, evident in all four types of interventions, although most clearly in community-based interventions, related to women’s need for psychological safety and the role of trustful relationships in helping women feel safe. Several studies documented how interventions created situations in which women were able to develop relationships of trust with those who supported them. This could be though one to one relationships or by the interventions creating safe spaces in which women could express themselves and feel secure.

The nature of the staff and volunteers supporting women, both their personal qualities and their roles were important to women facilitating them to develop trustful relationships. In Akhavan and Lundgren’s [27] study, midwives working with women and the doulas who supported them linked the ideas of trust to feelings of safety.

*‘To have someone with her who she trusts and can communicate with …is incredibly important and makes her feel safe’* [27].

Haugaard [32] similarly noted how a doula’s ongoing relationship with a woman and the continuity this provided allowed women to feel safe. One noted.

*‘when I arrived and spoke the same language as her and told her that I would be with her throughout the period—during childbirth and at the maternity ward—she felt safe. It was very important for her’* [32].

Brookes and Coster noted how staff who were seen to *be ‘friendly, caring approachable responsive and knowledgeable’* allowed women to ‘*forge strong relationships… and to build a high level of trust’* [28].

In addition to the personal characteristics of the individuals providing support their status or role was important. The value placed on the nonprofessional status of befrienders and peer supporters was evident in several interventions. Women felt that the befrienders were more available and less official than paid staff and that their relationship with them was therefore *'different from a midwife or health visitor more like a friend'* [35]. Interestingly, Ahmed’s study of breastfeeding peer supporters found that while women saw non-professionals as the key source of support their families and social networks did not, suggesting a difference in perception between the women who received support and their wider family/networks [33].

For some women trust and confidence in the individuals supporting them was also enhanced if that individual had similar experiences to them as they felt they were better placed to understand their needs [29, 31, 35]. In Hesselink’s study the shared language and culture of the Turkish community health workers helped women to overcome the ‘*the lack of interest and trust’* that had previously characterised relationships with health professionals, allowing a more positive engagement with services. In another situation a HIV positive woman who had been unwilling to take the advice of a consultant about her care, was able to trust this information when her befriender (who she knew shared her HIV status) reinforced the consultant’s advice [14].

A number of interventions functioned to provide, or facilitate access to, shared spaces in which women felt safe. One described how her befriender’s support in the local Children’s Centre as such a place.

*‘having someone to talk to at a children’s centre where I felt safe made all the difference, she saved me’* [14].

For some women, the sense of safety in a group was engendered by those delivering the intervention. They ensured that women felt that they were trustworthy and that they could ‘*keep confidence’*. This meant women felt safe with them and in the group they facilitated, something they did not feel in interactions with other professional services, noting that *‘it’s only in this group you can ask’* [30]. Other interventions acknowledged the importance of confidentiality in creating a safe and trusting environment for women but also highlighted some difficulties over the ways in which women understood ideas of more formal confidentially and statutory requirements for information sharing in some settings [28, 37].

Building trusting relationships with individuals supporting them facilitated women to confide in them and to access services they had previously been unable or unwilling to access for example, disclosing issues such as domestic abuse which meant women could then be supported to access the appropriate support [14].

### Practical Knowledge and Learning

A third theme related to the opportunity for learning and the acquisition of new knowledge. This was primarily, though not exclusively, linked to community-based interventions. It included women learning about medical issues related to pregnancy and birth, birthing and parenting including topics such as infant development, infant communication and discipline [28, 31, 32, 34]. Some women described how they valued the opportunities for learning that came from the intervention and the kind of practical advice and knowledge they gained,

*‘I love her so, so, sooooo much! She give me lots of advice, lots of, every week when she comes,’ another that they learned ‘how to do baby massage … [and where] learning lots of useful things’* [30].

Lederer [35] and Bhavnani and Newburn [29] found that the information about maternity care and support provided by their community-based interventions, meant that more women knew about what care and support was available to them as a result of the intervention. In addition to this increase in knowledge linked to the intervention, Lederer [35] noted an increase in women accessing services and community-based activities such as those at children’s centres. These and other studies linked new knowledge women gained to improvements in the perinatal experiences of the women, families and babies [14, 28, 34, 36, 38].

The studies by Brookes and Coster [28] and O’Shaughnessy et al. [30] found some of the parents they supported were unfamiliar with the cultural practices of the reception countries relating to birth and parenting, e.g. fathers being present at birth, feeding, sleeping and disciplining practices. One participant noted the differences with reception counties legislation on children’s rights, physical chastisement and FGM.

*‘This is what to do, that what you are doing is not good, do this and do this. So you said that and it’s changed an idea or opinion* [comment made in relation to smacking/disciplining *infants*]’ [30]

They noted how for some participants, particularly recently arrived people, the interventions led to changes in beliefs. One woman described how the intervention had affected her,

*‘In Africa, when you have the operation (Caesarean section), your family and friends cry and pray because there is a strong chance that you could die, so I was terrified and refused to have it, but on Baby Steps they managed to convince me that it is different here and that I would be alright’* [28].

There was also evidence that some interventions changed attitudes towards the practise of FGM and gender roles within relationships [28, 30] One woman noted,

*‘Before, I wasn’t allowed to go out and pick up the children from school or to go to the supermarket but now I can go and my husband stays home and looks after the children. I’m really grateful for that. He has also been helping me more and more with the housework and if I’m tired he takes over, I’m really happy about it’* [28].

In some of the interventions, gaining knowledge took the form of a mutually beneficial knowledge exchange. Midwives in Sweden explained how ‘*we learned from each other*’ [27] and the organisers of the Birth and Beyond intervention in the UK noted how those involved in providing the intervention ‘*learned a lot about working with marginalised and disadvantaged groups’* from the women they worked with [29].

In addition, there was a profound learning experience for many of those providing the interventions. This involved them gaining a deeper understanding of the material and physical challenges faced by asylum-seeking and refugee women in the perinatal period. This new knowledge meant that interventions had to be adapted to better reflect the experiences of asylum-seeking and refugee women and to meet their specific needs. Brookes and Coster [28] noted how ‘*the complex problems’* and additional challenges women faced, which included inadequate housing, insecure immigration status, relocation and severe financial hardship, meant that it was harder for them to take part in their intervention than they had expected and that ‘*a degree of tailoring was necessary in order to respond to these parents additional needs*’ [28]. It also meant that in some cases the.

*‘support provided by the practitioners was in fact far broader than the actual remit of Baby Steps’* [28].

In addition to the work they had planned to do, staff also acted as advocates, wrote letters to the home office and solicitors, liaised with other agencies, visited those who had been relocated and provided basic supplies to those in need, services that one woman described as ‘*a lifeline*’ [28]. Bhavnani and Newburn [29] adapted the nature of their interventions noting that ‘*support for these families [asylum-seeking and refugee] has often taken a more practical form’* and that this support was.

*‘more useful and culturally more understood and accepted among asylum-seekers and refugees, compared with contact for no purpose beyond talking listening and emotional support’* [29].

Bhavnani and Newburn also noted how they changed their recruitment policy from seeking volunteers who were exclusively asylum-seeking and refugee women to include women from different communities. This was because they learnt that some asylum-seeking and refugee women were not emotionally or practically ready to act as volunteers as ‘*they were often highly pressured by having to deal with their own asylum issues’* [29].

### Being Cared for and Emotional Support

A number of studies highlighted the role played by the interventions in providing care and emotional support [27–29, 35]. This support is particularly evident in the interventions characterised as community, community doula and paraprofessional.

The women in these studies spoke of experiencing depression, stress, anxiety, anger and of ‘*feeling scared’* as they faced pregnancy, birth and new parenthood in challenging situations [28, 29, 33, 35]. Bhavnani and Newburn [29] and Lederer [35] reported that that their interventions reduced levels of depression and anxiety in the women they supported with 15% less women reporting depressive symptoms following the intervention and 80% of women reporting a positive impact on their mood. Lederer [35] reported 59% of women (which included asylum-seekers and refugees in their sample) who were followed up had a reduced score for depression and 88% a reduced score for anxiety on the Hospital Anxiety and Depression (HAD) scale. Rocheron [37] found that for women continuity of care and emotional support were linked with reduced stress levels and that this seemed to reduce fears of hospital, labour and birth.

*‘if they weren’t around I would have felt scared… they were very helpful and comforting [link workers]’* [38].

Other women noted how they received emotional support from the project on woman saying that it gave ‘*support with my feelings as well as with the baby’s crying’* [35].

Having a place in which to express concerns to people who would listen to them was an important aspect of the support provided by the interventions. Brookes and Coster [28] noted how women felt, ‘*reassured by spending time and sharing experiences with other parents-to-be’* and that the intervention provided them with ‘*a forum for them to raise their concerns and worries*’ [28].

The importance of talking and being listened to was crucial to women. One woman noted that her befriender was *‘all ears wide’* another that.

*‘I am able to talk about my worries … I feel I have known her for 5 years, she understands me’* [35].

Another important aspect of emotional support provided by the interventions was the sense of being cared for by staff, peer supporters and doulas [28, 29, 33]. One woman explained that they ‘*made me feel like somebody actually wanted to help, somebody actually cared…’* [28].

### ‘I can do anything now’: Increased Confidence in and beyond the Intervention

The final theme, evident in community befriending/peer support and community befriending plus interventions was the positive impact of the interventions on the confidence of women (and families), both during and after the intervention. This increased confidence also included the community befrienders many of whom were facing similar challenges to the women they supported. In this way some interventions had an impact well beyond their remit of supporting specific women in the perinatal period.

A number of studies note how the interventions increased women’s confidence in their *ability to speak out in some contexts* [14]. The increased confidence gained from the interventions for other women related to their ability to overcome challenges related to birth and parenting. One woman noted that support helped her overcome her fears and give birth naturally to a 10 lb cephalic presented baby and that following that she felt ‘*I can do anything now’*. Other women reported increased confidence in their ability to parent and to communicate with and care for their babies [28, 30, 35]. Women felt more confident in accessing services and other sources of support that they had previously felt unable to [29, 35]. Beyond this confidence in their daily lives some women went on to take on new roles. A number of women involved in one project went on to act as.

*‘propagating keys’, by providing advice on maternity related issues, access to healthcare services and spreading health messages to their social circle’* [31 p. 57].

Lederer notes that many women felt ‘*more confident and are motivated and empowered to take control of their lives’* [35]. Brookes and Coster felt that for some the intervention was *‘transformative’* changing their lives and relationships [28]. Interventions also helped some women move forward with their lives, with some women moving from being supported to being supporters and others looking towards taking courses at college [14].

The positive impact on the self-esteem and self-confidence of volunteers working on the projects was noted in a number of studies [14, 29, 35]. One woman noted that ‘*It has made me aware of skills that I never thought I had. I feel strong’* [35]. Another that,

*‘I used to think I was nothing now I think I’m something and when I wear my refugee council badge I feel like a professional’* [14].

For some volunteers the projects provided an opportunity for progression into other voluntary roles or paid work, indeed Bhavnani and Newburn [29] argue that the community based nature of the project benefits social engagement for these volunteers as well as a wider sense of community connectedness and awareness.

## Discussion

The aim of this review was to explore existing literature on the provision, nature and impact of social support interventions on the wellbeing of asylum-seeking and refugee women in the perinatal period across Europe. This review identified four main types of intervention; educational, community befriending/peer support (and community befriending plus), community doula and paraprofessional support which took place at various points in the perinatal period. Analysing the nature of support offered insight into the differing rationales underpinning the interventions and into the ways in which asylum-seeking and refugee women were perceived.

The interventions helped to address the challenges that asylum-seeking and refugee women experience in the perinatal period. These include material challenges such as housing, finance and insecure legal status [4, 6, 7] as well as the psychosocial challenges associated with pregnancy, birth and new motherhood, including social isolation, lack of social support, poor mental health [4, 6]. Educational interventions were largely based on ideas of clinical need, risk, or perceived deficiencies in health behaviour. They sought to promote individual behaviour change rather than explicitly locating women within their social/cultural/political context or identifying structural or social issues which may contextualise some of these behaviours [24, 25, 36, 37]. Paraprofessional interventions sought to address the deficiencies in communication or culturally competent care within existing services [26, 33, 38]. In contrast, community befriending/peer support (and community befriending plus) interventions sought to address the needs of local communities by using resources from within those communities. The women supported are understood to be located within the legal, economic, social and gender structures of the reception county. The interventions focus on trying to help women to deal with these challenges and are predicated on a belief in the efficacy of befriending and volunteering in improving experiences of care, aspects of public health and reducing some health inequalities [14, 29, 30, 35]. In a similar way community doula support is located within and uses resources from, the community in which it arises to address the existent structural challenges faced by asylum-seeking and refugee women [27, 32, 34].

In order to understand the impacts of different interventions on the wellbeing of asylum-seeking and refugee women these interventions were mapped against the experiences of asylum-seeking and refugee women. This showed that all the interventions had some impact on some aspects of asylum-seeking and refugee women’s experience of the perinatal period. However, it was those based within the community; community befriending/peers support & community befriending plus and the community doula models that had the most positive impacts on women’s experiences and provided the most consistent support across the perinatal period. The review found that these interventions helped ‘alleviate feelings of being alone’. By developing trustful relationships and creating safe places they provided women with an increasing ‘sense of safety and trust’ as they accessed perinatal care. These interventions also provided women with ‘practical knowledge and learning opportunities’ as well as providing women with a sense of ‘being cared for and emotionally supported’. This review suggests only when these multiple levels of need are met by interventions that women start to feel empowered in the way encapsulated in the final theme ‘I can do anything now.’ The efficacy of the community-based befriending/peer support and doula models aligns with wider literature on the positive impact of community befriending/peer support on the wellbeing of groups lacking social support [39].

The way in which the community-based befriending/peer support interventions successfully address many aspects of asylum-seeking and refugee women’s needs throughout the largest part of the perinatal period, along with the underlying rationale of this type of intervention suggests this type of intervention may be most beneficial, via its holistic approach, to providing support for women. This aligns with research by Haith-Cooper and Bradshaw [40] and Rogers [41] who both highlight the importance of a holistic and socially located model of health for migrant and refugee women which places ‘ the pregnant women with the global context’ reflecting on the wider global, macro and micro contexts with in which women experience the perinatal period in their new country as well as need for psychosocial and practical support for women to help them access health systems [41].

## Strengths and Limitations

The strengths of this review are its European wide perspective and its inclusion of a wide range of peer and grey literature including qualitative, quantitative and mixed method approaches. Using relevance as a quality criterion, as part of the CIS approach, allowed access to studies that would be missed in some more traditional reviews, again expanding the scope of the review. The limitations of this study are that pragmatically only literature in English was used. There was a lack of clarity in some studies over participants country of origin, time in reception country and legal status which makes detailed analysis more challenging and tends towards the homogenisation of women’s experiences. Additionally, as found in previous research there were issues with definitions of asylum-seeking and refugee women, making data selection challenging as it can be hard to disaggregate data that is reported in more general categories.

## Implications for Practice and Research

This review suggests that further research is needed to explore the impact of specific social support interventions on the experiences of asylum-seeking and refugee women in different contexts and perinatal time periods. This research should be done in a collaborative way with the women these interventions are aimed to support. This research needs to avoid the over homogenisation of women’s experiences by ensuring that detailed data is collected including women’s country of origin, time in reception country and legal status as recommended by Balaam et al. [22].

Practitioners designing social support interventions for asylum-seeking and refugee women should take a more holistic view of their situation and needs in order to accommodate their complex and unique circumstances. Interventions should be developed with and using the experiences of the women they seek to support, taking a woman centred approach rather than designing interventions based on other actors’ perceptions of need as these interventions are more likely to meet women’s needs in a more effective way.

## Conclusion

This review explored existing literature on the provision, nature and impact of social support interventions on the wellbeing of asylum-seeking and refugee women in the perinatal period across Europe. The findings demonstrate that asylum-seeking and refugee women face a range of unique challenges in the perinatal period and that social support interventions have been developed to provide social support in this period. These interventions take a number of approaches, reflecting differing rationale’s and attitudes to asylum-seeking and refugee women’s needs, however the interventions most valued by women and which seem to have the most positive impact on women’s experiences are community-based befriending/peer support. Future work is needed to explore with women how to provide social support that most effectively meets their needs in the perinatal period to allow them to experience optimal wellbeing in the perinatal period and provides their infants with a positive start in life.

## Footnotes

1 A Doula is a lay individual who provides support for a woman in the perinatal period, particularly during birth.
